# Robotic Partial Cystectomy and Extended Pelvic Lymph Node Dissection for Node-Positive Urachal Adenocarcinoma in a 34-Year-Old Woman: A Case Report

**DOI:** 10.3390/curroncol33040190

**Published:** 2026-03-30

**Authors:** Stefanie Herrmann, Christian Gilfrich, Stephan Siepmann, Julio Ruben Rodas Garzaro, Fabian Eder, Stephan Schleder, Philipp Aubele, Felix Keil, Matthias May, Anton Kravchuk

**Affiliations:** 1 Department of Urology, Merciful Brothers Hospital St. Elisabeth, 94315 Straubing, Germany; stefanie.herrmann@klinikum-straubing.de (S.H.); christian.gilfrich@klinikum-straubing.de (C.G.); stephan.siepmann@klinikum-straubing.de (S.S.); julio.rodas-garzaro@klinikum-straubing.de (J.R.R.G.); anton.kravchuk@klinikum-straubing.de (A.K.); 2 Institute of Pathology, Hospital Deggendorf, 94469 Deggendorf, Germany; dr.f.eder@pathologie-deggendorf.de; 3 Department of Diagnostic and Interventional Radiology, Merciful Brothers Hospital St. Elisabeth, 94315 Straubing, Germany; stephan.schleder@klinikum-straubing.de; 4 Department of Hematology and Oncology, Straubing Hospital Medical Care Center, 94315 Straubing, Germany; philipp.aubele@mvz-klinikum-straubing.de; 5 Institute of Pathology, University of Regensburg, 93053 Regensburg, Germany; felix1.keil@klinik.uni-regensburg.de

**Keywords:** urachal adenocarcinoma, rare genitourinary malignancy, pelvic lymph node dissection, minimally invasive surgery, precision oncology, multidisciplinary tumor board, adjuvant chemotherapy, molecular profiling

## Abstract

Urachal carcinoma is a rare and aggressive malignancy for which standardized management remains limited, particularly in patients with locally advanced and node-positive disease. We report the case of a 34-year-old woman with urachal adenocarcinoma involving the bladder dome and radiographically suspicious pelvic lymph nodes who underwent robot-assisted partial cystectomy with urachal resection and extended bilateral pelvic lymph node dissection. Because there was no clinical, radiologic, or intraoperative evidence of umbilical involvement, the umbilicus was preserved after preoperative counseling and intraoperative confirmation of a negative proximal margin. Final pathology demonstrated a 4.5 cm enteric-type urachal adenocarcinoma, pT3a pN2 (2/17), with lymphovascular invasion, perineural invasion, and negative surgical margins. Immunohistochemistry and DNA- and RNA-based next-generation sequencing showed microsatellite stability, mismatch-repair proficiency, low tumor mutational burden, and no actionable genomic alteration. Given the absence of an established adjuvant standard, the multidisciplinary tumor board selected adjuvant FOLFOX as a non-standard postoperative strategy based on the overall clinicopathologic context. The patient remained continent, experienced no postoperative complications or treatment-limiting toxicity, and showed normalization of carcinoembryonic antigen and carbohydrate antigen 19-9 levels. This case provides a carefully contextualized example of transparent surgical reasoning and restrained multidisciplinary management in a rare malignancy with limited prospective evidence.

## Simple Summary

Urachal carcinoma is a rare and aggressive malignancy for which standardized management remains limited, particularly in patients with locally advanced and node-positive disease. We report the case of a 34-year-old woman with urachal adenocarcinoma who underwent robot-assisted partial cystectomy with urachal resection and extended bilateral pelvic lymph node dissection. The report highlights three clinically relevant aspects: a bladder-preserving robotic surgical approach with negative margins, a case-specific and explicitly justified decision to preserve the umbilicus in the absence of suspected involvement, and a structured postoperative multidisciplinary evaluation integrating histopathology, immunohistochemistry, and next-generation sequencing. Because no established adjuvant standard exists for urachal carcinoma, the postoperative treatment strategy required individualized decision-making. This case is presented not as a precedent-setting constellation, but as a carefully contextualized example of multidisciplinary management in a rare malignancy with limited prospective evidence.

## 1. Introduction

Urachal carcinoma (UrCA) is a rare malignancy arising from vestigial allantoic remnants along the urachal tract and accounts for fewer than 1% of bladder malignancies. Contemporary registry-based meta-analyses estimate an incidence of approximately 0.04 new cases per 100,000 person-years [1,2]. UrCA most commonly presents in the fifth or sixth decade of life, shows a consistent male predominance, and is frequently diagnosed at a locally advanced or metastatic stage, reflecting both its aggressive biologic behavior and the persistent lack of standardized diagnostic and therapeutic pathways [1,2,3,4,5,6,7].

Over the past decade, collaborative registry analyses, institutional series, and systematic reviews have substantially refined the clinicopathologic characterization of this disease. A comprehensive systematic review and meta-analysis including nearly 2000 patients confirmed a characteristic pattern of presentation, most commonly involving tumors of the bladder dome or anterior wall with extravesical extension and reported a 5-year overall survival of approximately 50% despite aggressive local therapy [2]. Across available datasets, surgery remains the cornerstone of management, most commonly in the form of partial cystectomy with resection of the urachal tract, whereas positive surgical margins and nodal involvement have repeatedly been associated with inferior oncologic outcomes [2,5,7,8,9].

At the same time, the pathologic and biologic framework of UrCA has become more nuanced. Consensus recommendations from the International Society of Urological Pathology and the current World Health Organization classification emphasize that diagnosis and staging require integration of the morphology, radiology, operative findings, and clinicopathologic context, while also underscoring the need to distinguish UrCA from primary bladder adenocarcinoma and secondary glandular malignancies involving the bladder [10,11,12]. Histologic subtype may also carry prognostic relevance, as recent population-level analyses suggest markedly worse outcomes for signet-ring variants than for non-signet-ring urachal adenocarcinoma [13].

Molecular profiling has further expanded the biologic understanding of UrCA, although its immediate therapeutic implications remain limited in most cases. Genomic and tumor microenvironment studies have demonstrated recurrent alterations in key oncogenic pathways and have suggested greater biologic overlap with colorectal adenocarcinoma than with urothelial carcinoma [4]. These observations have contributed to the use of colorectal-type systemic regimens in selected clinical scenarios, but they do not establish a validated therapeutic standard, particularly in the adjuvant setting [2,4]. Likewise, expression of potential antibody–drug conjugate targets such as Trop-2, Nectin-4, HER2, or Claudin 18.2 has generated translational interest, but these findings currently remain hypothesis-generating and do not yet support routine clinical application in UrCA [14].

Despite these conceptual advances, optimal local and postoperative management remains incompletely defined. The extent of lymph node dissection is not standardized; robotic experience is limited to small series and case reports; and prospective evidence to guide adjuvant treatment is lacking [2,15,16,17,18]. In this setting, carefully contextualized case reports remain valuable when they document clinically relevant decision-making with sufficient pathologic, surgical, and postoperative detail [2,4,5,8,9,10,12,14,15,16,17,18,19].

The present report describes a 34-year-old woman with node-positive urachal adenocarcinoma treated by bladder-preserving robotic surgery and subsequent multidisciplinary postoperative evaluation, with particular focus on the rationale for umbilical preservation, the extent of lymph node dissection, and the restrained integration of molecular findings into adjuvant treatment planning.

## 2. Case Presentation

### 2.1. Diagnosis, Preoperative Work-Up, and Surgical Management

A previously healthy 34-year-old woman presented with intermittent, painless gross hematuria that had persisted for three months. Clinical examination and abdominal palpation were unremarkable. Diagnostic cystoscopy revealed a suspicious lesion at the bladder dome. Transurethral resection was performed, and histopathologic evaluation of the specimen demonstrated an adenocarcinoma consistent with urachal origin, provisionally classified as high-grade/poorly differentiated on transurethral sampling, measuring 4.5 cm in greatest dimension and staged at least pT2.

**Figure 1:**
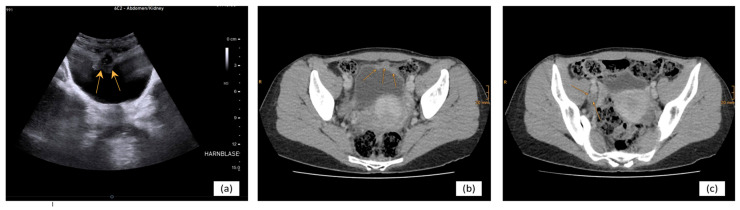
Preoperative cross-sectional imaging: Abdominal ultrasound performed prior to transurethral resection of the bladder demonstrates a well-defined mass at the bladder dome ((**a**), marked with arrows). Representative contrast-enhanced computed tomography images show a tumor arising from the urachal tract with extension into the bladder dome ((**b**), marked with arrows). A markedly enlarged lymph node is visible in the right pelvic region between the external iliac and obturator nodal stations, consistent with radiologically suspected nodal metastasis ((**c**), marked with arrows). No evidence of distant metastatic disease was identified on staging imaging.

Preoperative staging with contrast-enhanced computed tomography of the chest, abdomen, and pelvis demonstrated a tumor arising from the urachal tract with extension into the bladder dome, while maintaining a wide radiographic distance from the umbilicus (Figure 1a,b). In addition, enlarged and morphologically suspicious iliac lymph nodes were identified, including a centrally necrotic node located between the right external iliac and obturator nodal stations (Figure 1c). No evidence of distant metastatic disease was detected. Serum tumor markers were elevated, with a carcinoembryonic antigen level of 6.4 ng/mL and a carbohydrate antigen 19-9 level of 134 IU/mL [20].

Five days after histologic confirmation, the case was discussed by a multidisciplinary tumor board. Because the disease appeared locally advanced but surgically resectable, and because pelvic nodal involvement was suspected radiographically, surgical treatment with curative intent was recommended. In view of the patient’s age and the location of the tumor at the bladder dome, a bladder-preserving robotic approach was favored. The planned procedure therefore consisted of robot-assisted partial cystectomy with urachal resection and extended bilateral pelvic lymph node dissection.

The extent of umbilical resection was discussed explicitly with the patient before surgery. Because there was no clinical or radiologic evidence of umbilical involvement, a conditional strategy of umbilical preservation was adopted, with the understanding that the final decision would depend on intraoperative findings and margin assessment.

Surgery was performed without delay using a four-arm da Vinci Xi robotic system (Intuitive Surgical Inc., Sunnyvale, CA, USA). The patient was placed in steep Trendelenburg position. Trocar placement was shifted cranially relative to the umbilicus to optimize exposure of the superior bladder compartment and the urachal region, and an additional 12 mm assistant port was placed laterally on the right side. The camera port was positioned approximately 6 cm cranial to the umbilicus using an Alexis port (Applied Medical, Rancho Santa Margarita, CA, USA), allowing intact specimen retrieval in an endoscopic retrieval bag and access for frozen-section assessment when required.

**Figure 2:**
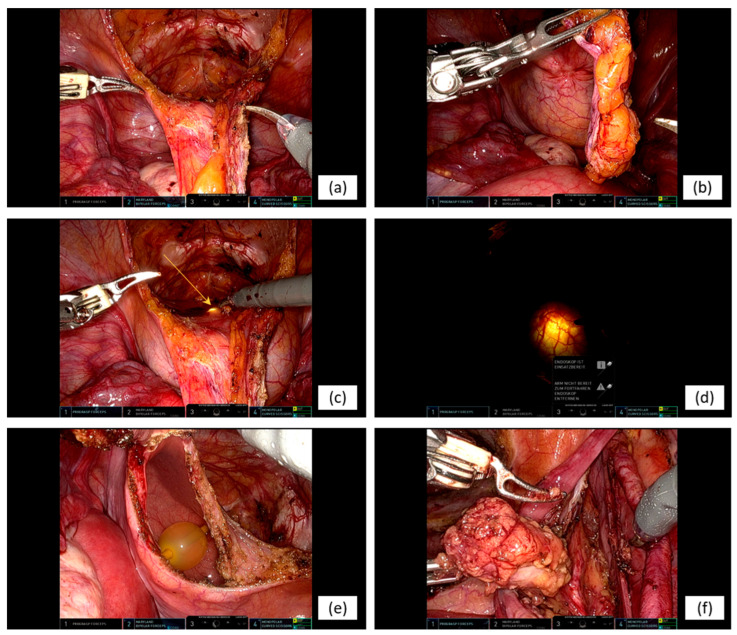
Intraoperative robotic surgical setting: Intraoperative views during robot-assisted partial cystectomy with extended PLND. The images illustrate dissection of the retropubic space (**a**); precise mobilization of the urachal tract (**b**); endoscopic delineation of bladder resection margins using flexible cystoscopy (**c**,**d**); full-thickness excision of the bladder dome lesion (**e**); and en bloc PLND with exposure of the right iliac vessels (**f**).

**Figure 3:**
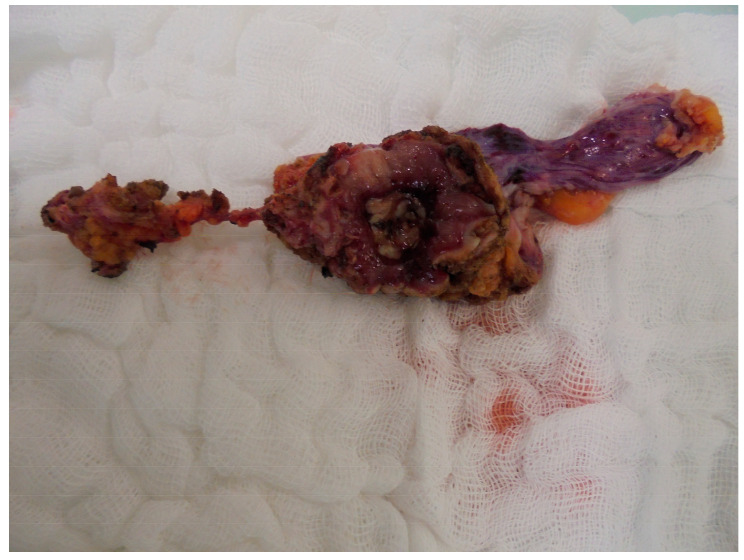
Resected surgical specimen: Macroscopic appearance of the specimen following robotic en bloc resection, including the involved bladder dome segment and the urachal tract. Complete tumor excision was achieved with intact surgical margins.

After entry into the retropubic space, the urachal tract was mobilized cranially and transected at the level of the umbilicus. Intraoperatively, no macroscopic evidence of umbilical involvement was observed. A flexible cystoscope was then introduced into the bladder using a cut-to-light technique to define circumferential resection margins, aiming for an approximate safety margin of 2 cm around the lesion. Full-thickness excision of the involved bladder dome segment was subsequently performed, followed by two-layer closure of the bladder defect (Figure 2a–f). Watertight reconstruction was confirmed intraoperatively by retrograde filling with 250 mL of saline. The specimen, including the involved bladder dome and urachal tract, was retrieved intact (Figure 3).

Extended bilateral pelvic lymph node dissection was then carried out, including systematic removal of the obturator, external iliac, and common iliac nodal stations up to the level of ureteral crossover. The enlarged lymph node identified preoperatively in the right obturator fossa was completely excised with preservation of the obturator nerve. Hemostasis remained meticulous throughout the procedure. A 14-Fr drain was placed into the space of Retzius via the left trocar site. The operation was completed without intraoperative complications. Estimated blood loss was 25 mL, total operative time was 180 min, and console time was 115 min.

Postoperative recovery was uneventful. The patient remained hemodynamically stable, laboratory values stayed within normal limits, and oral intake, full mobilization, and the first bowel movement occurred on postoperative day 1. A cystogram performed on postoperative day 6 demonstrated preserved bladder integrity without evidence of extravasation. After catheter removal, the patient achieved complete bladder emptying without residual urine or upper urinary tract dilatation, with voided volumes of up to 250 mL.

**Figure 4:**
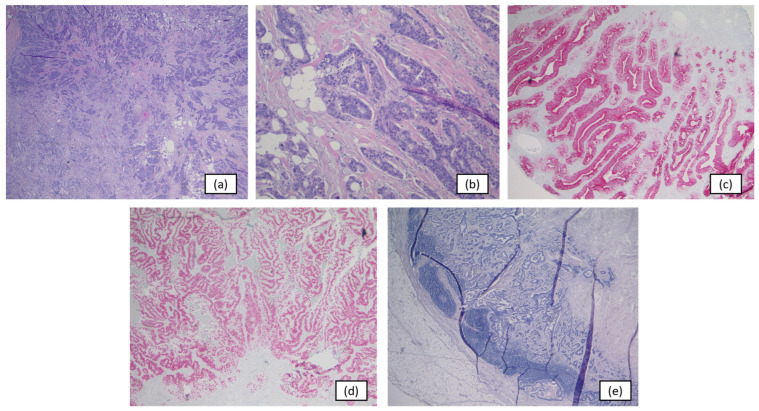
Histopathology and Immunohistochemistry: Representative histological sections illustrating adenocarcinoma consistent with urachal carcinoma. The predominant tumor component, removed during transurethral resection, demonstrated poor differentiation, whereas the residual tumor identified in the definitive surgical specimen showed moderate differentiation. Immunohistochemical staining reveals an enteric differentiation pattern. The panels show adenocarcinoma at 20× (**a**) and 100× magnification (**b**); cytokeratin 20 positivity at 40× magnification (**c**); CDX2 positivity at 40× magnification (**d**); and a metastatic lymph node at 20× magnification (**e**).

Final histopathologic examination confirmed a locally advanced, moderately differentiated enteric-type adenocarcinoma without mucinous or signet-ring cell components (Figure 4). The tumor was classified as pT3a, grade 2, with a residual maximum diameter of 15 mm. Surgical margins were negative, with a circumferential clearance of 5 mm toward the bladder lumen, at least 1 mm relative to adjacent structures including the peritoneal surface, and 10 cm toward the umbilical side. Lymphovascular invasion and perineural invasion were present. Regional lymph node assessment demonstrated metastatic involvement in 2 of 17 nodes, corresponding to pN2 disease according to the eighth edition of the TNM classification for urinary bladder cancer [10,21]. According to the Sheldon and Mayo classification systems, the tumor was staged as IVa and III, respectively [7,22]. All findings were subsequently reviewed in the postoperative multidisciplinary tumor board. Two weeks after surgery, serum carcinoembryonic antigen and carbohydrate antigen 19-9 levels had declined to within the normal range.

### 2.2. Postoperative Multidisciplinary Evaluation and Adjuvant Therapy Decision-Making

Postoperatively, the case was re-evaluated by a dedicated multidisciplinary tumor board integrating the operative findings, final pathologic staging, and extended immunohistochemical and molecular characterization. Given the presence of locally advanced, node-positive urachal carcinoma (pT3a pN2) with lymphovascular and perineural invasion, the risk of early systemic relapse was considered substantial.

**Figure 5:**
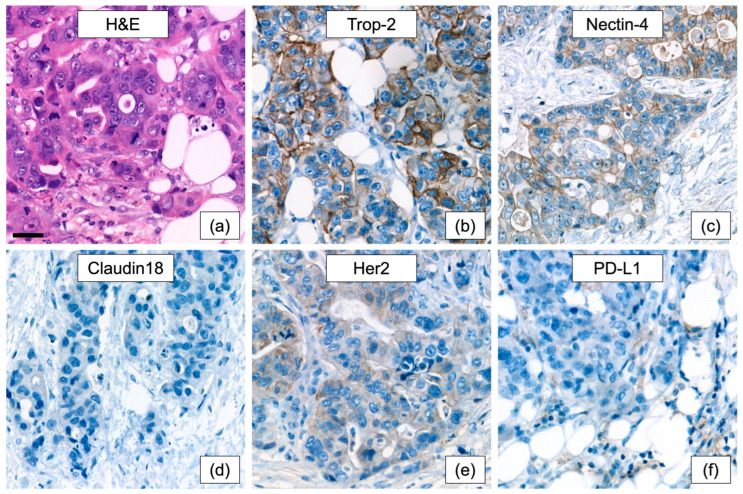
Predictive immunohistochemical analyses: H&E (**a**) and immunohistochemical stains (**b**–**f**) are shown at 200× magnification (see scale bar equating 50 µm in (**a**)). Trop-2 showed moderate expression (**b**) with an H-score of 185, while Nectin-4 showed low-level expression (**c**) with an H-score of 65. No expression of Claudin 18 (**d**) was identified, while HER2 showed low (1+) expression (**e**). Only few tumor-associated immune cells were PD-L1-positive, while the tumor cells were negative (**f**).

Because no evidence-based adjuvant standard exists for urachal carcinoma, additional molecular work-up was performed to document baseline tumor biology, exclude plausible but unsupported biomarker-driven alternatives, and support a more informed postoperative discussion in a setting defined largely by retrospective data and biologic extrapolation rather than prospective trials [2,23,24,25]. DNA- and RNA-based next-generation sequencing was performed using the TruSight Oncology 500 panel (TSO500; Illumina, San Diego, CA, USA) to assess single-nucleotide variants, copy-number alterations, gene fusions, tumor mutational burden (TMB), homologous recombination deficiency (HRD), and microsatellite status. Microsatellite instability was additionally assessed by polymerase chain reaction-based fragment analysis and by immunohistochemical evaluation of mismatch-repair protein expression (MLH1, PMS2, MSH2, and MSH6). Immunohistochemical studies included the standard molecular tumor board panel with assessment of HER2 and PD-L1, supplemented by Trop-2, Nectin-4, and Claudin-18. The results are summarized in Table 1 and illustrated in Figure 5. Overall, the tumor showed microsatellite stability, low TMB, no actionable genomic alteration, low-level Nectin-4 expression, moderate Trop-2 expression, and a TP53 mutation. These findings did not identify a validated biomarker-based adjuvant strategy and did not support escalation toward immunotherapy or targeted treatment in the postoperative setting [4,12,14,26,27]. Accordingly, potential consideration of antibody–drug conjugates such as enfortumab vedotin or sacituzumab govitecan was discussed only as a hypothetical future option in the event of recurrent or disseminated disease, not as a treatment implication for the adjuvant setting. In addition, genetic counseling was recommended to exclude a germline TP53 alteration.

The postoperative systemic treatment decision was, therefore, made on the basis of the overall clinicopathologic context rather than as a direct consequence of molecular profiling. In the absence of an established adjuvant standard for urachal carcinoma, and considering the enteric differentiation pattern, nodal positivity, lymphovascular invasion, and lack of a more compelling evidence-based alternative, the multidisciplinary tumor board selected a fluorouracil-, leucovorin-, and oxaliplatin-based regimen (FOLFOX) as a non-standard, case-specific adjuvant strategy. This decision was informed by the recognized biologic overlap between urachal adenocarcinoma and colorectal adenocarcinoma, while explicitly acknowledging that such overlap does not constitute prospective evidence for adjuvant benefit in urachal carcinoma [2,4,23,24,25,28,29]. Historical cisplatin-based regimens were also considered as part of the broader literature on systemic treatment in urachal carcinoma, particularly in advanced disease; however, no regimen has been established as a uniform postoperative reference standard [2,5,6,23,25]. Given the patient’s age, potential treatment-related fertility implications were discussed, and oocyte cryopreservation was performed prior to initiation of systemic therapy. Port placement and referral to medical oncology were arranged to enable timely initiation of systemic treatment.

### 2.3. Early Follow-Up

The patient completed four of the twelve planned biweekly cycles of adjuvant FOLFOX without postoperative complications, significant toxicity, or treatment-limiting adverse events. Urinary continence was preserved, and no impairment of sexual function was reported. Serum carcinoembryonic antigen and carbohydrate antigen 19-9 levels remained within the normal range [20]. Restaging after completion of six cycles is planned and will include magnetic resonance imaging of the pelvis and abdomen as well as computed tomography of the chest. Formal quality-of-life data were not prospectively collected. Accordingly, the present report provides only early postoperative and early adjuvant follow-up, and mature oncologic outcome data are not yet available.

## 3. Discussion

Urachal carcinoma remains one of the most challenging rare malignancies in genitourinary oncology because of its low incidence, frequent presentation at advanced stage, and the persistent absence of prospective trials to guide evidence-based management [1,2,3,5,6,7]. Population-based and multicenter analyses have consistently shown that adverse pathologic features, nodal involvement, and incomplete resection are associated with inferior oncologic outcomes, whereas carefully contextualized individual cases continue to retain practical value in areas where standardized algorithms remain limited [2,3,5,7,8,9,30]. The present report does not rest its relevance on the novelty of any single component, but on the transparent documentation of a clinically instructive convergence of locally advanced node-positive disease, bladder-preserving robotic surgery, case-specific surgical decision-making, and restrained postoperative treatment planning in a rare malignancy for which prospective evidence remains scarce.

From a surgical perspective, this case supports the technical feasibility of robot-assisted partial cystectomy with urachal resection and extended pelvic lymph node dissection in carefully selected patients treated at experienced centers. Current robotic data remain limited to small series and case reports, and no meaningful conclusions regarding superiority over open surgery can be drawn from the available literature [15,16,17,18]. The value of the present case therefore lies not in suggesting comparative advantage, but in showing that a robotic approach can achieve negative margins, low blood loss, and an uncomplicated early postoperative course even in the setting of radiographically suspicious nodal disease. These observations should be interpreted descriptively and within the context of a still heterogeneous evidence base [2,18].

The decision to preserve the umbilicus deserves explicit comment. Classical surgical dogma has long favored complete excision of the urachal tract together with umbilectomy, a paradigm historically traceable to Begg’s anatomic description from 1930 [31]. In the present case, however, there was no clinical, radiologic, or intraoperative evidence of umbilical involvement. The issue was discussed preoperatively with the patient, who specifically questioned the oncologic value of routine resection of a macroscopically uninvolved umbilicus, particularly in the context of nodal disease. Intraoperatively, the urachal tract was transected at the umbilical level, and final pathology demonstrated a negative proximal margin exceeding 10 cm toward the umbilical side. Importantly, more recent literature suggests that omission of umbilectomy is not without precedent. In the comprehensive systematic review and meta-analysis by Suartz et al., umbilical management could be specifically assessed in 948 patients, and the umbilicus was preserved in 360 of these evaluable cases, corresponding to 38.0% [2]. Although these data do not establish oncologic equivalence, they do indicate that routine umbilectomy in the absence of suspected umbilical involvement remains incompletely evidence based. We, therefore, consider umbilical preservation in this case to represent a deliberate individualized oncologic decision rather than a broadly generalizable principle.

The extent of lymph node dissection similarly requires cautious interpretation. In urachal carcinoma, neither the indication for pelvic lymph node dissection itself nor the optimal nodal template is supported by a uniform evidence base. In the systematic review by Suartz et al., pelvic lymph node dissection was explicitly documented in only 377 of 1640 surgically treated patients, and the extent of dissection was inconsistently reported [2]. In addition, the recent stage-stratified analysis by Zhu et al. further illustrates ongoing heterogeneity in real-world nodal practice [32]. In our patient, extended bilateral pelvic lymph node dissection was chosen because of radiologically suspicious regional pelvic nodes and the desire to optimize regional staging and local-regional cytoreduction. By contrast, presacral and para-aortic dissection was not undertaken because those nodal basins were radiologically uninvolved and because no evidence demonstrates that prophylactic extension into nonregional nodal fields improves oncologic outcomes in urachal carcinoma [2,33]. This restraint was also informed by the broader oncologic principle that wider nodal templates may increase morbidity, a concern that remains relevant even in bladder cancer, where lymphadenectomy has been studied far more extensively yet still remains debated with respect to optimal extent and treatment burden [34,35]. In the present case, avoiding an oncologically unproven extension of dissection beyond the radiologically involved pelvic field was considered the most appropriate balance between staging, cytoreduction, and recovery, particularly because timely initiation of adjuvant systemic therapy was an important postoperative objective.

The postoperative systemic treatment decision also required careful calibration. No evidence-based adjuvant standard exists for urachal carcinoma, and available systemic treatment strategies remain derived largely from retrospective reports, broader reviews, and biologic extrapolation [2,6,23,24,25]. Cisplatin-based combinations have historically been used, particularly in advanced disease, while colorectal-type regimens have been adopted in selected cases because urachal adenocarcinoma shares morphologic and, in part, genomic features with colorectal adenocarcinoma [23,24,25,29]. The recent multicenter phase II study of modified FOLFIRINOX is important in this context, but it was conducted exclusively in recurrent or metastatic disease and cannot be extrapolated to the adjuvant setting [28]. In our patient, the choice of adjuvant FOLFOX was therefore made as a pragmatic, non-standard multidisciplinary decision based on nodal positivity, enteric differentiation, and the absence of a more compelling evidence-based alternative, rather than as a treatment strategy validated by the molecular profile itself. The molecular analyses were valuable primarily as a descriptive baseline characterization and as a means of excluding unsupported therapeutic assumptions. The tumor was microsatellite stable, TMB-low, and devoid of actionable alterations, and therefore did not provide a persuasive rationale for adjuvant immunotherapy or targeted treatment [4,12,14,26,36]. Likewise, serum tumor markers were considered useful for longitudinal monitoring rather than for directing postoperative treatment selection [20].

Several limitations should be acknowledged. First, this is a single case and cannot support generalized recommendations regarding robotic management, umbilectomy, nodal templates, or adjuvant therapy. Second, follow-up remains limited to the early postoperative and early adjuvant course, and mature oncologic outcome data are not yet available. Third, no formal quality-of-life data were prospectively collected. Within these limits, the present report offers a clinically contextualized case study that emphasizes transparent surgical reasoning, restrained interpretation of molecular findings, and multidisciplinary decision-making in a rare malignancy for which prospective evidence remains scarce.

## 4. Conclusions

This case illustrates the management of locally advanced, node-positive urachal adenocarcinoma through a carefully individualized multidisciplinary strategy that combined bladder-preserving robotic surgery, explicit case-specific reasoning regarding umbilical preservation and lymph node dissection and restrained postoperative systemic treatment planning informed by detailed pathologic and molecular characterization. The report does not support broad recommendations regarding robotic superiority, routine omission of umbilectomy, nodal template extension, or adjuvant regimen selection. Rather, it highlights the importance of transparent clinical judgment in a rare malignancy for which prospective evidence remains scarce and major management decisions continue to rely on multidisciplinary interpretation of incomplete data. Within these limits, the present case may offer practical value for clinicians facing similarly complex presentations of urachal carcinoma.

## Figures and Tables

**Table 1:** Comprehensive Next-Generation Sequencing and Immunohistochemical Profiling of the Tumor and Clinical Interpretation for Adjuvant Decision-Making in Urachal Carcinoma.

Biomarker/Pathway	Method	Result	Clinical Interpretation	Potential Therapeutic Relevance
**MMR/MSI status**	PCR-based microsatellite analysis; IHC (MLH1, PMS2, MSH2, MSH6)	Microsatellite stable; preserved nuclear expression of mismatch repair proteins	Mismatch repair-proficient tumor biology	No biological rationale for immune checkpoint inhibition
**Tumor mutational burden (TMB)**	NGS (522-gene panel)	0.0 mutations/Mb (low)	Molecularly “cold” tumor profile	No biological rationale for immune checkpoint inhibition
**Homologous recombination deficiency (HRD)**	NGS-based genomic instability score	Negative; GIS 27	Intact DNA double-strand break repair capacity	No rationale for PARP inhibitor-based approaches
**Mutation analysis**	NGS (522-gene panel)	TP53 pathogenic missense mutation c.742C>T/p.R248W (NM_000546.6) (allele fraction 50%)	Disruptive DNA-binding domain mutation	Potential prognostic relevance only; no approved targeted therapy
**Gene fusions/splice variants**	NGS (RNA-based fusion analysis)	None detected	No targetable rearrangements identified	No role for fusion-directed therapies
**Copy number variations (CNV)**	NGS	Low-level copy number gains involving MYC, FGFR1, ERBB3, KRAS, and MYCL	Subclonal or low-amplitude alterations without established predictive relevance	No validated indication for targeted therapy
**PD-L1**	IHC (Dako, clone 22C3)	TPS 0%; IC 1%; CPS 2	Absence of tumor cell PD-L1 expression	No biological rationale for immune checkpoint inhibition, but no established cut-off values for UrCA type
**HER2 (ERBB2)**	IHC (Dako, rabbit polyclonal)	low (Rüschoff-score 1+)	No HER2-driven tumor biology	No sufficient evidence for HER2-targeting therapy in UrCA
**Trop-2**	IHC (Abcam plc., clone SP294)	Moderate expression (H-score 185)	Antigen present at intermediate level without an established predictive threshold in UrCA	No biological rationale for Trop-2-ADC therapy
**Nectin-4**	IHC (Abcam plc., clone EPR15613-68)	Low expression (H-score 65)	Antigen present at low level without an established predictive threshold in UrCA	No biological rationale for Nectin-4-ADC therapy
**Claudin-18**	IHC (LifeSpan BioSciences)	0% membranous expression	Target antigen absent	No biological rationale for Claudin 18.2-targeting therapy

**Footnote**: Comprehensive next-generation sequencing was performed using a 522-gene panel within a multidisciplinary molecular tumor board framework. The resulting molecular profile is presented as a descriptive baseline characterization of tumor biology. None of the detected genomic alterations or immunohistochemical findings currently provide a validated rationale for targeted or immunotherapeutic treatment in the adjuvant setting of urachal carcinomas. Any potential relevance in recurrent or metastatic disease remains hypothetical and would require dedicated clinical validation. **Abbreviations**: ADC = antibody–drug conjugate; CNV = copy number variation; CPS = combined positive score; ERBB = erb-B receptor tyrosin kinase; FGFR1 = fibroblast growth factor receptor 1; GIS = genomic instability score; HER2 = human epidermal growth factor receptor 2; HRD = homologous recombination deficiency; IC = immune cells; IHC = immunohistochemistry; KRAS = kirsten rat sarcoma viral oncogene homolog; Mb = megabase; MLH = MutL homolog; MMR = mismatch repair; MSH = MutS homolog; MSI = microsatellite instability; MYC = myelocytomatosis oncogene; MYCL = MYC proto-oncogene; NGS = next-generation sequencing; PARP = poly (ADP-ribose) polymerase; PCR = polymerase chain reaction; PD-L1 = programmed death-ligand 1; PMS = postmeiotic segregation increased 2; RNA = ribonucleic acid; TMB = tumor mutational burden; TP53 = tumor protein p53; TPS = tumor proportion score; Trop-2 = trophoblast cell surface antigen 2; UrCA = urachal carcinoma.

## Data Availability

The original contributions presented in this study are included in the article. Further inquiries can be directed to the corresponding author.

## Abbreviations

The following abbreviations are used in this manuscript:

ADC	antibody–drug conjugate
CDX2	caudal type homeobox 2
CNV	copy number variation
CPS	combined positive score
DNA	deoxyribonucleic acid
ERBB	erb-B receptor tyrosin kinase
FGFR1	fibroblast growth factor receptor 1
FOLFIRINOX	leucovorin, 5 fluorouracil, irinotecan and oxaliplatin chemotherapy regimen
FOLFOX	fluoropyrimidine and oxaliplatin chemotherapy regimen
Fr	French
GIS	genomic instability score
H&E	hematoxylin and eosin
HER2	human epidermal growth factor receptor 2
HRD	homologous recombination deficiency
H-Score	histochemical score
IC	immune cells
IHC	immunohistochemistry
KRAS	Kirsten rat sarcoma viral oncogene homolog
Mb	megabase
MLH	MutL homolog
MMR	mismatch repair
MSH	MutS homolog
MYC	myelocytomatosis oncogene
MYCL	MYC proto-oncogene
NGS	next-generation sequencing
PARP	poly (ADP-ribose) polymerase
PCR	polymerase chain reaction
PD-L1	programmed death-ligand 1
PLND	pelvic lymph node dissection
PMS	postmeiotic segregation increased
RNA	ribonucleic acid
TMB	tumor mutational burden
TNM	tumor node metastasis
TPS	tumor proportion score
TP53	tumor protein 53
Trop-2	trophoblast surface antigen 2
UrCA	urachal carcinoma
